# *Trichinella pseudospiralis*-secreted 53 kDa protein ameliorates imiquimod-induced psoriasis by inhibiting the IL-23/IL-17 axis in mice

**DOI:** 10.1016/j.bbrep.2022.101415

**Published:** 2022-12-27

**Authors:** Sukhonthip Khueangchiangkhwang, Zhiliang Wu, Isao Nagano, Yoichi Maekawa

**Affiliations:** a Department of Parasitology and Infectious Diseases, Gifu University Graduate School of Medicine, Gifu, Japan; b Preemptive Food Research Center, Gifu University, Gifu, Japan

**Keywords:** *Trichinella pseudospiralis*, Excretory-secretory protein, Psoriasis model, IL-23/IL-17 axis

## Abstract

*Trichinella* infection can experimentally ameliorate many autoimmune diseases. However, the immune mechanism of the amelioration and the identification of corresponding *Trichinella*-derived molecule(s) are still not fully elucidated. Fifty-three kDa excretory-secretory (ES) protein from *Trichinella pseudospiralis* (Tpp53) is a molecule like TsP53 reported as a protein exerting immune-inhibitory effect in *T. spiralis*. In this study, we investigated the immunomodulatory effect of Tpp53 using imiquimod (IMQ)-induced psoriasis-like dermatitis model, which is a mouse model of autoimmune disease with the pathogenic interleukin 17 (IL-17) producing CD4^+^ T cells (Th17) via IL-23/IL17 axis. Administrating the recombinant Tpp53 (rTpp53) mixed with IMQ cream on the skin of mice ameliorated psoriatic lesions, as revealed by the improvement of erythema, scaling, skin thickening, epidermis hyperplasia and parakeratosis, thickening of acanthosis cell layer, epidermal extension of dermis, less infiltration of inflammatory cells, and decreased expression of inflammatory marker. The increased expression of the factors related to the IL-23/IL-17 axis, including IL-17A, IL-6, Il17F and Il23a, in the skins of IMQ-treated mice was inhibited by rTpp53 treatment. Moreover, the expression of activated keratinocyte-produced cytokines, chemokines, and antimicrobial peptides in the skin was also down-regulated in rTpp53-treated IMQ-treated mice. Co-culture of splenocytes with rTpp53 inhibited IL-17A and treatment of macrophages with rTpp53 reduced IL-6 production. Overall, our study revealed that the *Trichinella*-secreted 53 kDa ES protein could ameliorate IMQ-induced psoriasis by inhibiting the IL-23/IL-17 axis, suggesting that Tpp53 might involve in regulating host Th17 for immune evasion and have an alternative potential for psoriasis therapy.

## Highlights

• First-time utilization of imiquimod (IMQ)-induced psoriasis-like dermatitis in the study of immunomodulation by parasite-derived molecules.
• Recombinant *Trichinella pseudospiralis*-secreted 53 kDa ES protein (rTpp53) could ameliorate IMQ-induced psoriasis.
• rTpp53 inhibited the IL-23/IL-17 axis, keratinocyte-produced cytokines, chemokines and antimicrobial peptides in the psoriatic skin lesions.
• rTpp53 suppressed Th17 response of splenocytes and IL-6 production from intraperitoneal macrophages in vivo and *in vitro.*

## Graphical abstract

**Figure:**
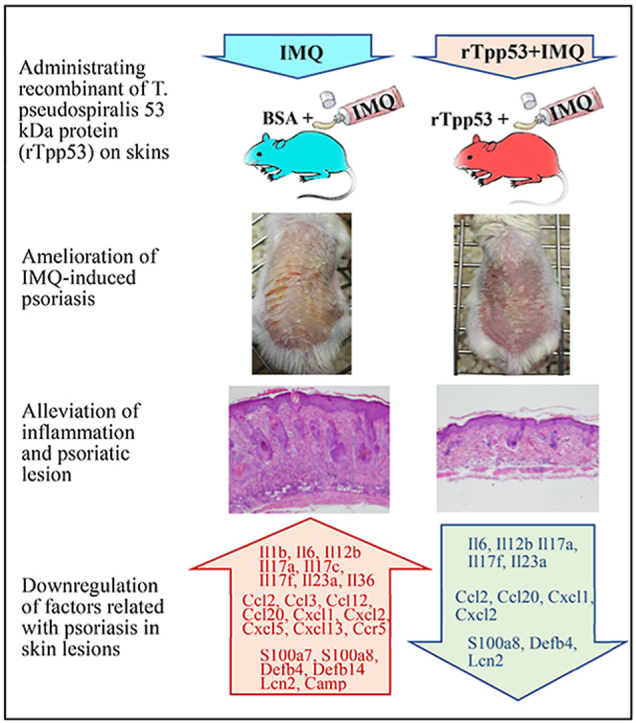

## 1 Introduction

*Trichinella* is a parasite that can survive in the host for the long term by immune evasion. *Trichinella* is the largest intracellular parasite and can survive for a long period of time in a muscle niche. The mechanism of immune evasion in *Trichinella* infection has been widely studied using experimental models of autoimmune and allergic diseases, for example, experimental autoimmune encephalomyelitis (EAE) [1,2], type 1 diabetes [3], TNBS-induced experimental colitis [4], collagen-induced arthritis [5,6], and allergic inflammation models [7]. Although understanding of the mechanism is still limited, studies have revealed several potentials, including evoking dominant T helper (Th) 2 immunity to attenuate the Th1 and Th17 responses [2,8] and inducing regulatory T cells [9], regulatory B cells [10], regulatory dendritic cells [11] and alternatively activated macrophages (M2 macrophages) [12].

Identification of *Trichinella*-derived molecules is critical in defining the immunomodulatory mechanism of these infections. Excretory-secretory (ES) products of *Trichinella* are regarded to be important in modifying the host’s immune response. Studies have indicated that ES of *T. spiralis* can inhibit the production of proinflammatory cytokines of macrophages, such as TNF-α and IL-6 [13,14], activate M2 macrophages [15,16], and promote the production of anti-inflammatory cytokines, such as IL-10 and TGF-β [17,18]. Several ES proteins have been identified to be immunomodulatory, such as *T. spiralis*-derived paramyosin (TsPmy) [19], serine protease inhibitors (Ts-SPIs) [20], cathepsin B-like protein (TsCPB) [21], and the 53 kDa ES protein (TsP53).

TsP53, characterized in 1990 [22], is a 53 kDa ES protein from *T. spiralis*, a encapsulated species of *Trichinella*. There is no homologue to be found in GenBank, and its function is still unknown. However, recent studies have revealed that TsP53 has immunomodulatory functions. Recombinant TsP53 (rTsP53) protected mice from colitis associated with upregulated Th2 and Treg responses [16,23,24]. The rTsP53 attenuated lipopolysaccharide (LPS)-induced damage of endotoxaemia [25] and provided protective effects against acute lung injury in mice [26] by activating and promoting M2 macrophage polarization. Tpp53 (53 kDa ES protein from *Trichinella pseudospiralis*) is the same kind of ES protein as TsP53. *T. pseudospiralis* is a non-encapsulated species of *Trichinella*. Besides the difference in encapsulation of cysts between the two species of *Trichinella*, *T. pseudospiralis* infection induces a stronger inhibition of inflammation than *T. spiralis* [[27], [28], [29]]*.* There is no report on the immunomodulatory function of the Tpp53, but it is supposed to be similar to TsP53.

Psoriasis is an immune-mediated cutaneous inflammatory disorder. It is characterized by a thickened epidermis because of hyperproliferative keratinocytes, acanthosis, parakeratosis, hyperkeratosis, and infiltration of inflammatory cells in psoriatic skin. Multidirectional communication between innate immune cells and resident skin cells such as neutrophils, dendritic cells (DCs), macrophages, neutrophils and keratinocytes, is involved in the pathogenesis of psoriasis [30]. These interactions result in the differentiation of adaptive immune cells, leading to histopathological features of psoriasis [31]. Innate cells such as DCs and neutrophils plays important roles in the development of psoriasis [32]. The stimulation causes keratinocytes to secret cytokines and chemokines which activate innate immune cells such as DCs and neutrophils [33]. Chronic skin inflammation in psoriasis is then induced by the cytokines, chemokines and oxidant mediators secreted from activated innate immune cells [34]. DCs locates in a critical position in the complex network of immune cells in the pathogenesis of psoriasis. Regulated by Bruton’s tyrosine kinase (BTK) signaling, the antigen-presenting cells connect the signaling cascade of innate and adaptive immunity through pro-inflammatory cytokines [32,35]. Therefore, the pathogenesis of psoriasis involves the crosstalk between keratinocytes and immune cells in a feed-forward way [36,37]. In this mechanism, stimulated keratinocytes function as the trigger of psoriasis by secreting cytokines, chemokines, and antimicrobial peptides (AMPs) to activate DCs which secret multiple proinflammatory cytokines such as TNF-α, interleukin (IL)-6, IL-12, IL-23 and IL-36. These cytokines in turn activate resident keratinocytes and infiltrating T cells, including IL-17-producing CD4^+^ T cells (Th17) which produce IL-17A, IL-17F, and other cytokines. Th17-produced cytokines further stimulate keratinocytes, fibroblasts, and innate immune cells to produce a variety of cytokines, chemokines, and AMPs, which promote the expansion of Th17 cells and amplify local inflammatory responses [38]. Thus, the crosstalk between keratinocytes, innate cells and Th17 cells drives cutaneous inflammation, keratinocyte proliferation and differentiation, and epidermal hyperplasia. The mouse model of imiquimod (IMQ)-induced psoriasis is a well-established animal model for psoriasis study and has been confirmed to resemble human psoriasis in the mechanism of pathogenesis and immunity, including a major involvement of the IL-23/IL-17A axis [39]. IMQ activates resident DCs and keratinocytes via TLR7 which play an important role in the development of autoimmune diseases such as psoriasis [40,41]. Therefore, this model is widely used in the study of immunity, pathogenesis, and therapy of psoriasis. Approaches in psoriasis therapy are mainly focused on inhibiting immune signaling such as TNF-α, IL-17, IL-12/IL-23 [42,43], BTK [32,35], and other tyrosine kinases [44].

Th17 cells play an important role in adaptive immunity protecting against pathogens and are involved in autoimmune disorders and inflammatory responses. Although our previous study showed that *Trichinella pseudospiralis* infection ameliorated EAE in a model of autoimmune disease by suppressing Th17 and Th1 responses [2], the molecule and mechanism responding to the inhibition of the Th17 response have not yet been elucidated. In the present study, we employed the model of IMQ-induced psoriasis to reveal the immunomodulatory function of Tpp53, a *Trichinella*-secreted molecule, on the Th17 response. By administering rTpp53 on the skin of the IMQ-induced psoriasis mouse, we investigated the effects of rTpp53 treatment on the alleviation of psoriasis, the expression of cytokines related to the IL-23/IL-17 axis in the psoriatic skin, the expression of keratinocyte-producing cytokines, chemokines and AMPs, and the direct effect on splenocytes and macrophages.

## 2 Materials and methods

### 2.1 Parasite and animal

*Trichinella pseudospiralis* (ISS13) was used in the present study. Eight-week-old female BALB/c were obtained from Japan SLC, Inc. All mice were maintained under pathogen-free conditions in individually ventilated cages with standard conditions (23 °C room temperature and 12:12 h light: dark cycle) at the Animal Resources Center of Gifu University Graduate School of Medicine. All animal care and experimental procedures were approved by the Committee for Animal Research and Welfare of Gifu University.

### 2.2 Preparation of recombinant 53 kDa ES protein of *T. pseudospiralis* (rTpp53)

rTpp53 was produced by the method previously described [45]. In brief, the gene encoding the pro-protein of the 53 kDa protein of *T. pseudospiralis* was amplified from muscle larvae cDNA. The PCR product was cloned into the plasmid vector pCold-GST (TAKARA, Tokyo, Japan).

The rTpp53 used in the skin administering was prepared using the *E. coli* expression system. The recombinant plasmid was transfected into competent *E. coli* (New England BioLabs, Beverly, MA, USA). rTpp53 was isolated and purified with a GSTrap HP column (GE Healthcare Bio-Sciences Co., Piscataway, NJ, USA). The purified recombinant protein was further treated with Detoxi-Gel™ Endotoxin Removing Gel (Thermo Scientific, Rockford, IL, USA) to remove endotoxins.

To avoid the potential effect of endotoxins when used in the *in vitro* experiment, rTpp53 was synthesized with a wheat germ cell-free protein expression system using a WEPRO7240G Expression Kit (Cell Free Sciences, Yokohama, Japan). The synthesized rTpp53 was purified and assessed as mentioned above.

### 2.3 IMQ-induced psoriatic-like mouse model

**Fig. 1:**
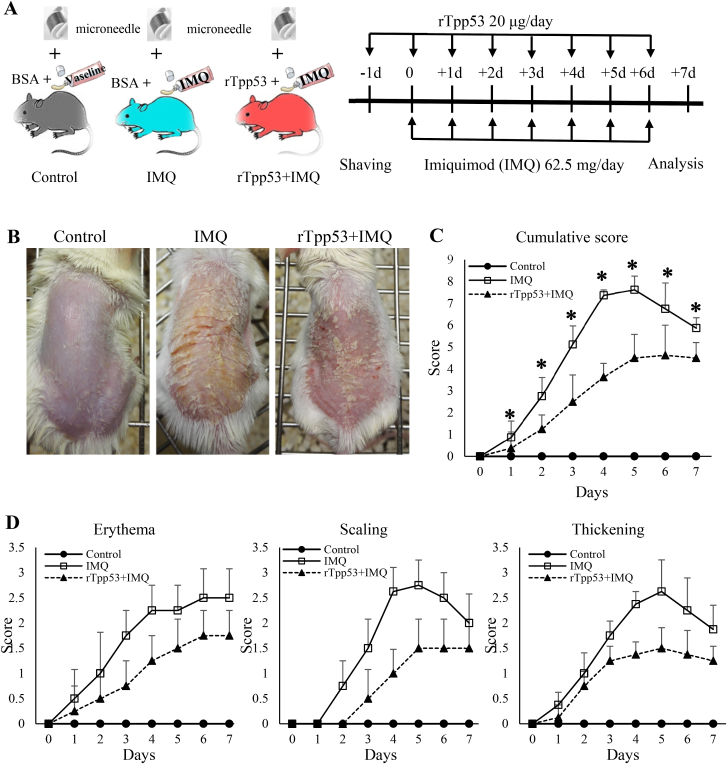
**rTpp53 ameliorated IMQ-induced psoriasis-like dermatitis in a mouse model**. (A) Flow chart of the experiment. Each group consisted of 5 mice. (B) Photos of back skin at 7 days post-IMQ induction. (C and D) PASI scores were evaluated daily, including erythema, scaling, thickening and cumulative scores. Animal group: Control: skins were smeared with Vaseline mixed with BSA; IMQ: skins were smeared with IMQ cream mixed with BSA; rTpp53+IMQ: skins were smeared with IMQ cream mixed with rTpp53. Data are expressed as the mean ± SD. * indicates P<0.05 between the IMQ and rTpp53+IMQ groups. The same experiments were repeatedly performed five times.

The study was not able to calculate the mouse number of cases based on power, as is used in clinical trials, because there was no information on effect sizes and no information for setting them up. Therefore, as shown in Fig. 1A, the number of mice was set at 5 per group, in accordance with the tradition of many previous animal studies. Fifteen mice were mechanically and randomly assigned to three groups: Control, IMQ and rTpp53+IMQ. The hair on the back skin at a surface area of approximately 2 × 2 cm was shaved, and 5% IMQ cream (Aldara, 3 M Pharmaceuticals, MN, USA) was applied daily at a dose of 62.5 mg for 7 days to establish an IMQ-induced psoriasis mouse model. To promote the absorption of rTpp53, the skin was rolled with a microneedle Derma Roller (1.0 mm) four times. Then, the mice in the control group were smeared with BSA (20 μg/20 μl) and Vaseline. The mice in the IMQ group were smeared with cream containing 62.5 mg IMQ mixed with 20 μl solution containing 20 μg BSA, and the mice in the rTpp53+IMQ group were smeared with cream containing 62.5 mg IMQ mixed with 20 μl solution containing 20 μg rTpp53. We chose this relatively high dose of rTpp53 because this was the exploratory study to examine whether rTpp53 had an inhibitory effect on IMQ-induced psoriasis.

### 2.4 Measurement of skin inflammation severity

The Psoriasis Area and Severity Index (PASI), consisting of measurements of skin erythema, scaling, and thickening, was used to evaluate skin inflammation in the skin lesions of mice. Briefly, erythema, scaling and thickening were each graded on a scale from 0 to 4 as follows: 0, none; 1, slight; 2, moderate; 3, marked; and 4, severe. The cumulative score served as a measure of inflammation severity (scale: 0–12). Mice were evaluated daily. The thickness of the back skin and ears of mice was measured using a micrometer.

To evaluate inflammatory cell filtration in the skin and measure epidermal thickness, back skin (0.5 × 0.5 cm) and ear tissues were paraffin-embedded, sectioned and stained with H&E. The thickness of the mouse epidermis was measured in 20 fields of view. MPO was measured using a Myeloperoxidase (MPO) Activity Assay Kit (Abcam).

### 2.5 Quantitative PCR (qPCR)

The expression of psoriasis-related cytokines, chemokines and AMPs in skin lesions was investigated with qPCR. Total RNA was isolated from the back skin (0.5 × 0.5 cm) and an ear that was ground thoroughly in 1 ml RNAiso Plus (TAKARA) in a homogenizer. The investigated genes and qPCR primers were designed based on the published sequences in GenBank, as shown in Supplementary Table 1. Reverse transcription was performed using a ReverTra Ace qPCR RT Kit (TOYOBO, Japan), and qPCR was performed using THUNDERBIRD Next SYRB qPCR Mix (TOYOBO). The relative quantity of the target mRNA was normalized to the level of the housekeeping gene *Gapdh*. The fold difference in the expression of target genes was determined using the comparative delta-delta CT method.

### 2.6 Effects of rTpp53 on splenocytes and intraperitoneal macrophages

Single-cell suspensions of spleens prepared from the mice in the control, IMQ and rTpp53+IMQ groups were cultured. The cells were stimulated with 100 ng/ml LPS (Sigma) or 1:1000 anti-CD3 (Biolegend). After 24 h of LPS stimulation or 72 h of anti-CD3 stimulation, the supernatants were collected, and IL-17A was measured with ELISA. In the experiment on the *in vitro* effect of rTpp53, the splenocytes and intraperitoneal macrophages from normal mice were cocultured with rTpp53 (10 μg/ml) for 12 h and then stimulated with LPS and anti-CD3. IL17A and IL-6 in supernatants were measured with ELISA.

### 2.7 ELISA

The back skin (0.5 × 0.5 cm) and the ear of the mouse were cut into small pieces and ground thoroughly in 1 ml PBS in a homogenizer to obtain the tissue suspension. The prepared homogenate was centrifuged at 15,000 g at 4 °C for 20 min, and the supernatant was taken for ELISA detection. Capture ELISA was performed to quantify IL-17A and IL-6 using reagents (BioLegend, San Diego, CA, USA).

### 2.8 Statistical analysis

All data are expressed as the mean ± SD. Statistical analysis was performed using an unpaired Student's *t*-test to compare intergroup differences. A value of p<0.05 was considered statistically significant.

## 3 Results

### 3.1 rTpp53 ameliorated IMQ-induced psoriasis-like dermatitis

To examine whether *T. pseudospiralis*-secreted 53 kDa excretory-secretory protein (Tpp53) can ameliorate the IL-17–mediated pathogenesis of psoriasis in vivo, we used an IMQ-induced psoriasis-like dermatitis model that is critically dependent on the IL-23/IL17 axis. Psoriasis was induced by IMQ cream mixed with or without rTpp53, as shown in Fig. 1A. All mice were shaved and rolled with a microneedle. Then, a 2 × 2 cm area in the back and both ears of the IMQ group were smeared with a mixture of BSA (20 μg) and IMQ (62.5 mg), and the group rTpp53+IMQ was smeared with a mixture of rTpp53 (20 μg) and IMQ (62.5 mg). The control group was treated with BSA and Vaseline. As shown in Fig. 1B, the mice of the IMQ group showed a typical psoriasis-like appearance on the back, which was improved in the rTpp53+IMQ group. All mice were evaluated with PASI, including erythema, scaling, and thickening. Compared with the IMQ group, the scores of erythema, scaling, thickening, and cumulative score were lower in the rTpp53+IMQ group (Fig. 1C and D).

### 3.2 rTpp53 inhibited the infiltration of inflammatory cells and the release of inflammation-related factors and improved IMQ-induced skin lesions

**Fig. 2:**
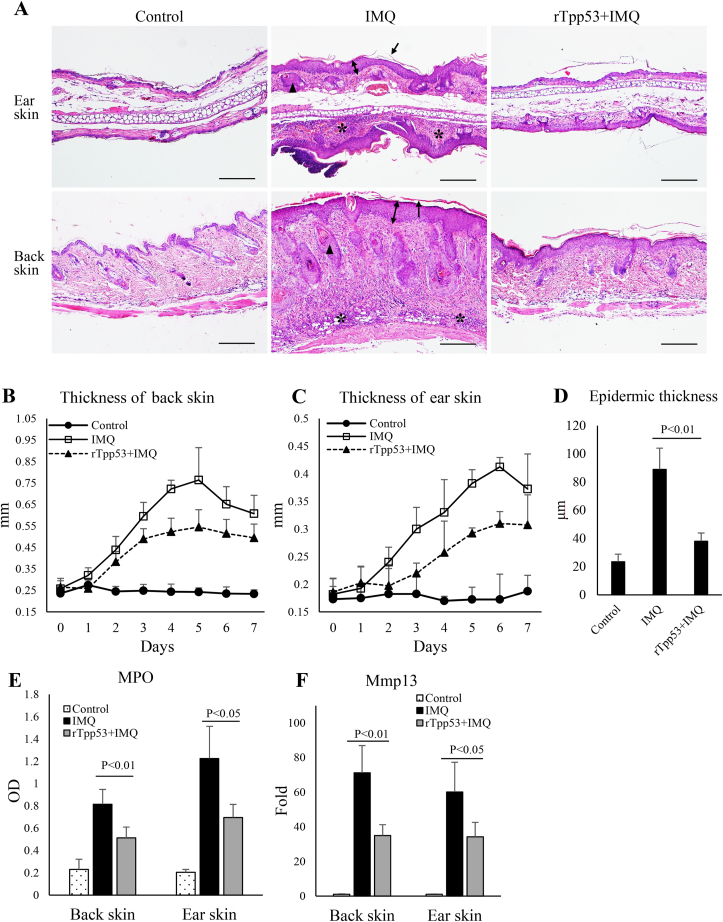
**rTpp53 inhibited inflammatory cell infiltration and inflammation-related factor release and improved IMQ-induced skin lesions**. (A) H&E staining of back and ear skins of mice (n=5) in Fig. 1A. Each photomicrograph is a representative image from every group. Single arrow: hyperkeratosis, double-sided arrow: acanthosis, black triangle: elongation of rete-like ridges, asterisk: inflammation infiltration, Bar=100 μm. (B and C) The thickness of back and ear skin measured daily. (D) The epidermal thickness of the back skin was measured from H&E-stained sections. (E) Expression of MPO in the back and ear skins. (F) Expression of *Mmp13* in the back and ear skin by qPCR. Data are expressed as the mean ± SD. The same experiments were performed at least three times.

Histological analysis of H&E staining of the back skin and ears indicated that rTpp53 treatment alleviated the severity of IMQ-induced skin psoriatic lesions. As shown in Fig. 2A, IMQ induced typical pathogenic skin psoriatic lesions in the IMQ group, including epidermal hyperplasia and parakeratosis, thickening of the acanthosis cell layer, and epidermal extension of the dermis. These kinds of psoriatic changes were greatly improved in the rTpp53+IMQ group. rTpp53 treatment caused less epidermal hyperplasia, as revealed by decreased thicknesses of the back and ear skin (Fig. 2B and C) and decreased epidermal thickness (Fig. 2D). There was excessive inflammatory cell infiltration in the dermis and epidermis in the IMQ group (Fig. 2A). Inflammatory infiltration was greatly inhibited in the rTpp53+IMQ group (Fig. 2A). The improvement in inflammation was also confirmed by the decreased expression of the inflammatory markers MPO and *Mmp13* in the rTpp53+IMQ group (Fig. 2E and F).

### 3.3 rTpp53 inhibited the expression of the IL-23/IL-17 axis in the psoriatic skins of IMQ-treated mice

**Fig. 3:**
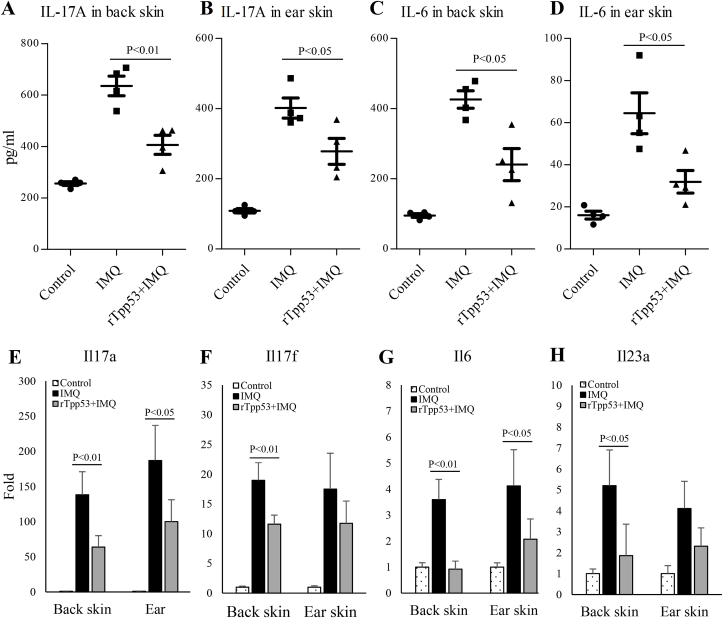
**rTpp53 inhibited the expression of the IL-23/IL-17 axis in the psoriatic skins of the IMQ-induced mouse model**. The protein expression levels of IL-17A in the back skin (A) and ear skin (B) and IL-6 in the back skin (C) and ear skin (D) from mice (n=5) in Fig. 1A were measured with ELISA. The mRNA expression levels of *Il17a* (E), *Il17f* (F), *Il6* (G) and *Il23a* (H) were measured with relative qPCR. Data are expressed as the mean ± SD. The same experiments were performed three times.

To investigate the effects of rTpp53 on the expression related to the IL-23/IL-17 axis, which is the main immunological mechanism of pathogenesis in psoriasis, the proteins and total RNA were isolated from the skins of the back and ear, and the protein expression levels of IL-17A and IL-6 were measured with ELISA. The mRNA expression levels of these cytokines and the genes related to the IL-23/IL-17 axis were determined with qPCR. IL-17A and IL-6 levels in the IMQ group were significantly higher than those in the control group (Fig. 3A–D). The increase in cytokines was inhibited by rTpp53 treatment, as there were significantly lower concentrations in the rTpp53+IMQ group than in the IMQ group (Fig. 3A–D). Correspondingly, the mRNA expression levels of *Il17a*, *Il17f*, *Il6* and *Il23* in the back and ear skins were increased in the IMQ group, while the increased expression was significantly suppressed in the rTpp53+IMQ group (Fig. 3E–H).

### 3.4 rTpp53 downregulated the expression of keratinocyte-producing cytokines, chemokines, and AMPs in the psoriatic skins of IMQ-treated mice

**Fig. 4:**
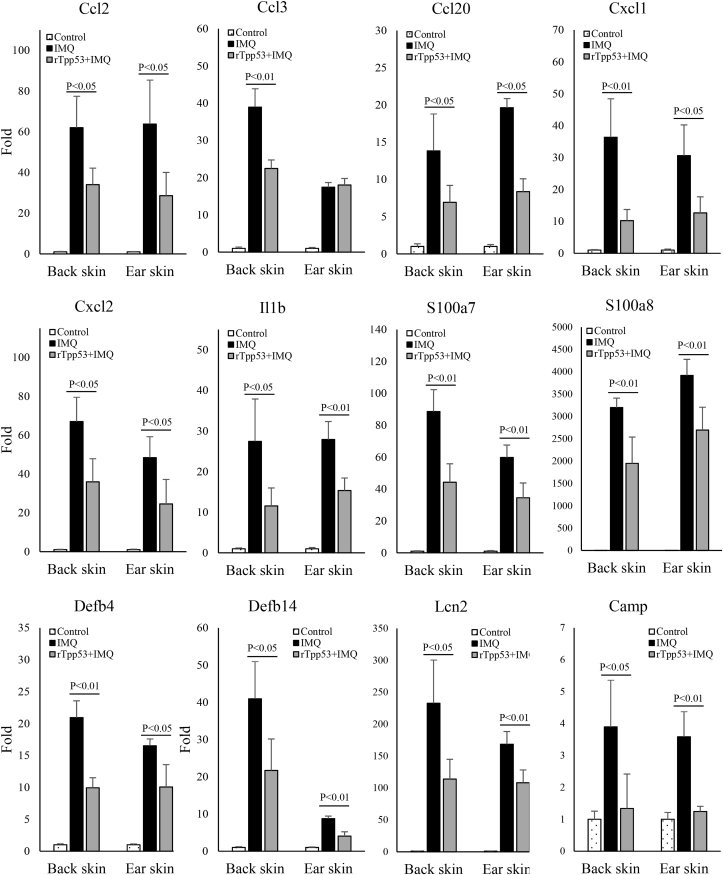
**rTpp53 downregulated the expression of keratinocyte-producing cytokines, chemokines, and AMPs in the psoriatic skins of the IMQ-induced mouse model**. Total RNA was isolated from the back skin and ear skin from mice (n=5) in Fig. 1A. The expression was measured with relative qPCR. Data are expressed as the mean ± SD. The same experiments were performed three times.

Upon stimulation and the effect of the feed-forward mechanism, keratinocytes in the skin produce various kinds of cytokines (Il36, Il1b, Ill17c, Il22, and Il8), chemokines (Ccl2, 3, 5, 17, 20, 22, and Cxcl1, 2, 5, 9, 10, 11), and AMPs (S100a7, S100a8, S100a9, Defb4, Defb14, Lcn2, and Camp), which play critical roles in the pathogenesis of psoriasis. Therefore, we investigated the expression of these factors. As shown in Fig. 4 and Table 1, there was increased expression of the cytokine *Il1b* in the IMQ group, and the increased expression was inhibited in the rTpp53+IMQ group. The expression levels of the chemokines *Ccl2*, *Ccl3*, *Ccl20*, *Cxcl1*, and *Cxcl2* were increased in the IMQ group, and the increased expression levels were inhibited in the rTpp53+IMQ group (Fig. 4 and Table 1). The expression of AMPs (*S100a7*, *S100a8*, *Defb4*, *Defb14*, *Lcn2* and *Camp*) was upregulated in the IMQ group, and rTpp53 treatment remarkably reduced the expression of these AMPs in the MIQ+rTpp53 group (Fig. 4 and Table 1).

**Table 1:** Expression of cytokines, chemokines, and antimicrobial peptides in the psoriatic skin lesions of IMQ-induced psoriasis mice and the effects by rTpp53.

**Gene**	**Expression relative to Control (fold)**						**Cellular origin in psoriatic lesion**	**Putative functions in psoriasis**
	**in back skin**			**in ear skin**				
	**MIQ**	**rTpp53 +IMQ**	**P value**	**MIQ**	**rTpp53 +IMQ**	**P value**		
*Ccl2*	63.85	34.01	0.07	63.78	28.59	0.03	Basal KCs	Th1 and Th17 cells trafficking
*Ccl3*	38.96	22.48	0.00	17.43	18.03	0.59	Epidermis, Upper dermis	Attracting Th1 cells, monocytes, and DCs
*Ccl12*	3.96	4.67	0.52	5.40	6.30	0.50	unknown	Attracting eosinophils, monocytes and lymphocytes
*Ccl20*	13.84	6.92	0.03	19.63	8.35	0.02	Suprabasal KCs	Th17 trafficking
*Cxcl1*	31.09	10.22	0.03	30.61	12.65	0.02	Suprabasal KCs	Th17 cells trafficking, KC hyperproliferation
*Cxcl2*	71.20	35.95	0.07	48.38	24.52	0.03	Basal KCs	Th17 cells trafficking, DC trafficking
*Cxcl5*	2.28	1.99	0.81	4.80	5.93	0.41	Basal KCs	Th17 trafficking, DC trafficking
*Cxcl9*	1.05	1.16	0.62	0.92	0.83	0.59	KCs, Macrophages	Th1 and pDC trafficking
*Cxcl10*	1.07	0.73	0.73	1.44	1.30	0.76	KCs	Th1 and pDC trafficking
*Cxcl13*	35.78	32.91	0.51	75.26	77.05	0.87	unknown	Chemotaxis of B cells
*Ccr5*	4.63	5.17	0.41	3.93	2.82	0.07	Basal KCs	Chemotaxis of eosinophils
*Il1b*	27.44	11.54	0.03	27.89	15.33	0.00	KCs, DCs	Th1 and Th17 trafficking
*Il6*	2.87	0.74	0.00	3.56	1.79	0.04	KCs, DCs	Th1 and Th17 trafficking
*Il12b*	8.54	3.24	0.01	13.41	5.07	0.03	DCs, macrophages	Th1 and Th17 cells trafficking
*Il17a*	138.29	63.90	0.01	187.05	100.10	0.03	Th17 cells	KC proliferation, proinflammatory
*Il17c*	6.56	4.83	0.36	3.91	4.92	0.22	KCs	Th17 cells trafficking
*Il17f*	18.98	11.59	0.004	17.50	11.75	0.16	Th17 cells	KC proliferation, proinflammatory
*Il23*	5.20	1.86	0.03	4.11	2.31	0.06	DCs, macrophages	Th17 cells trafficking
*Il36*	8.82	8.83	0.18	4.55	3.80	0.06	KCs	Attracting neutrophils, myeloid cells and T cells, induceing cytokines, chemokines, and AMPs
*Tnfa*	1.11	1.62	0.08	0.94	0.69	0.28	Th1 cells, DCs, macrophages	KC proliferation
*Mmp8*	18.13	16.74	0.81	14.70	16.74	0.42	unknown	unknown
*Mmp13*	71.20	34.94	0.005	60.17	34.23	0.03	KCs, fibroblasts	unknown
*S100a7*	88.58	44.27	0.003	59.75	34.50	0.006	KCs	Chemotaxis of neutrophils, and CD4^+^ T cells
*S100a8*	3200	1950	0.007	3919	2696	0.008	KCs	Chemotaxis of neutrophils, and CD4^+^ T cells
*Defb4*	20.93	9.94	0.000	16.53	10.06	0.012	KCs	Th17 trafficking, DC activation
*Defb14*	40.94	21.67	0.026	8.74	4.04	0.000	KCs	Th17 trafficking, DC activation
*Lcn2*	232.7	113.9	0.019	168.5	108.3	0.005	KCs, neutrophils	Inducing inflammation and epidermal proliferation
*Camp*	3.90	1.34	0.03	3.58	1.24	0.00	KCs	Activating various immune cells

Abbreviations: KCs: keratinocytes; Control: control group mice; MIQ: MIQ-induced psoriasis group mice; rTpp53+IMQ: rTpp53 treated MIQ.

### 3.5 rTpp53 suppressed IMQ-induced systemic immune responses in vivo

**Fig. 5:**
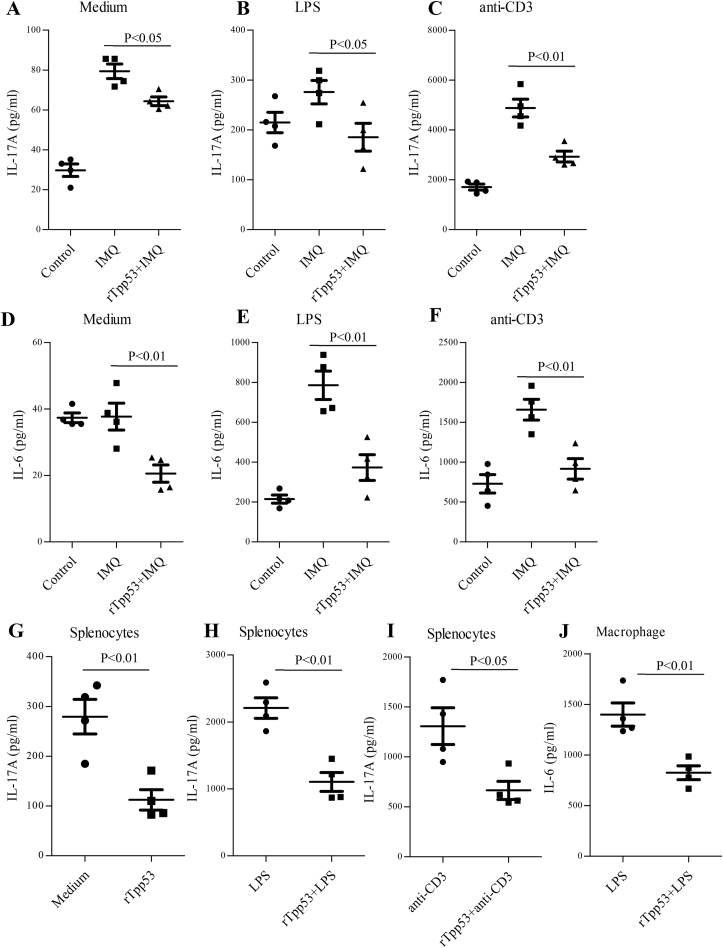
**rTpp53 suppressed IMQ-induced systemic immune responses in vivo and immune responses of splenocytes and intraperitoneal macrophages in vitro**. The splenocytes from the mice (n=5) in the control, IMQ and rTpp53+IMQ groups in Fig. 1A were cultured without stimulation (A, D) and stimulated with LPS (B, E) and anti-CD3 (C, F). In the *in vitro* experiment, the splenocytes and intraperitoneal macrophages from normal mice were cocultured with rTpp53 and then stimulated with LPS (H, J) and anti-CD3 (I). The cytokines IL-17A and IL-6 were measured with ELISA. A: IL-17A without stimulation; B: IL-17A with LPS; C: IL-17A with anti-CD3; D: IL-6 without stimulation; E: IL-6 with LPS; F: IL-6 with anti-CD3; G: IL-17A without stimulation; H: IL-17A with LPS; I: IL-17A with anti-CD3; J: IL-6 with LPS. Data are expressed as the mean ± SD. The same experiments were performed three times.

To observe whether rTpp53 treatment has effects on IMQ-induced systemic immune responses, the cells from the spleen at 7 days post-IMQ induction were cultured and stimulated with LPS or anti-CD3 *in vitro*, and the cytokines IL-17A and IL-6 were measured with ELISA. As shown in Fig. 5, the production of IL-17A in splenocytes was increased in the IMQ group under LPS (Fig. 5B) or anti-CD3 (Fig. 5C) stimulation and even without stimulation (Fig. 5A). All the increases in IL-17A production were suppressed in the rTpp53+IMQ group. Similarly, the production of IL-6 with LPS (Fig. 5E) or anti-CD3 (Fig. 5F) stimulation was increased in the IMQ group, and the increases were inhibited in the rTpp53+IMQ group.

### 3.6 rTpp53 inhibited the induction of IL-17A in splenocytes and IL-6 in macrophages *in vitro*

To assess whether rTpp53 directly affected the immune responses of splenocytes, mouse splenocytes were cocultured with cell-free synthetic rTpp53 and then stimulated with LPS or anti-CD3. As shown in Fig. 5G, even without stimulation, the production of IL-17A by splenocytes was inhibited by rTpp53 coculture. The rTpp53 coculture also inhibited the LPS-elevated or anti-CD3-elevated IL-17A production of splenocytes (Fig. 5H, I).

We also observed the *in vitro* effect of rTpp53 on macrophages. The intraperitoneal macrophages were cocultured with rTpp53 and then stimulated with LPS. As shown in Fig. 5J, the LPS-elevated IL-6 production was inhibited by rTpp53 coculture.

## 4 Discussion

In the present study, we investigated the immunomodulatory effect of a 53 kDa protein secreted from *Trichinella pseudospiralis* (Tpp53) on a Th17-related disease using an IMQ-induced psoriasis model. As a result, we found that the administration of recombinant Tpp53 (rTpp53) on the skin could ameliorate IMQ-induced psoriasis, as revealed by the improvement of pathological lesions of psoriasis (epidermis hyperplasia and parakeratosis, acanthosis, epidermal extension, and inflammatory infiltration), and the inhibited expression of IL-23/IL-17 axis related cytokines and chemokines in psoriasis skins.

The understanding of the mechanism by which parasitic helminths modify host immunity to escape host attack is still limited, especially regarding the parasite-derived molecules responsible for immunomodulation. The utilization of animal models of autoimmune and allergic diseases is a means to study the mechanism. Many approaches have been taken in studying *Trichinella* infection-induced immunomodulation, for example, EAE, type 1 diabetes, TNBS-induced experimental colitis, collagen-induced arthritis, and allergic inflammation models.

Little is known about the modulation of the Th17 response by *Trichinella*, and few *Trichinella*-derived molecules have been identified to be responsible for immunomodulation. In an animal model, IMQ induces psoriasis-like dermatitis, mainly via the IL-23/IL-17 axis. The IL-23/IL-17 axis is important in psoriasis pathogenesis because keratinocytes and infiltrating immune cells in psoriatic lesions constitutively express a receptor for IL-17 [46,47]. Therefore, we used a microneedle to open small pores on the skins of the back and ears and smeared them with a mixture of rTpp53 in IMQ cream to determine whether rTpp53 treatment can ameliorate IMQ-induced psoriasis and its immunological mechanism. The administration of rTpp53 could improve the severity of psoriasis, as revealed by lower PASI scores, less thickening of the skin, less inflammatory infiltration, and less epidermal thickening and parakeratosis.

Various kinds of cytokines are produced in psoriatic lesions and play important roles in the inflammation and development of psoriasis, especially the pivotal effector cytokines IL-23 and IL-17. IL-17 is a critical cytokine in the pathogenesis of psoriasis. In skin psoriasis, Th17-cell-produced IL-17A and IL-17F mainly act on keratinocytes to induce the production of various inflammatory mediators and facilitate the abnormal proliferation of keratinocytes [48]. When skin tissue receives stimulations, such as trauma, injury, infection, and medication, the stressed or damaged cells in prepsoriatic skins release autoantigens such as nucleic acids, cationic antimicrobial peptides/proteins (AMPs), ADAMTSL-5, and PLA2G4D, which activate DCs and then through IL-23 to activate Th17 cells [38]. DCs also produce the cytokines IL-6, IL-1β and TNF-α to further enhance Th17-cell differentiation from CD4^+^ cells, which produce high levels of IL-17A, IL-17F, IL-22 and TNF-α. All these cytokines act on keratinocytes to constantly produce proinflammatory cytokines, chemokines, and AMPs, which form a feed-forward mechanism to cause epidermal hyperproliferation and differentiation in the IL-23/IL-17A axis. Our present study showed the success of IMQ-induced psoriasis as a model to study the IL-23/IL-17A axis. There were obvious increases in the amount of IL-17A and IL-6 in the back skin and the expression of *Il17a*, *Il17f*, *Il6* and *Il23* in the skin of IMQ-treated mice; for example, the expression of *Il17a* was increased by 138-fold in the back skin of IMQ group mice. Treatment with rTpp53 decreased the amount of IL-17A and downregulated the expression of *Il17a* and *Il23*. Our previous study indicated that *T. pseudospiralis* infection decreased the expression of *Il17a*, *Il1b* and *Il6* in the pathological tissues and splenocytes of EAE model mice [2]. Some *Trichinella*-derived proteins have been confirmed to inhibit IL-17 production in PBMCs, such as succinate coenzyme A ligase beta-like protein (SUCLA-β) [49]. TsP53 has been confirmed to be anti-inflammatory by its suppressive effects on DCs and macrophages [16,26,50]. Therefore, the results of our study suggest that rTpp53 ameliorated psoriasis by downregulating the Th17 response, possibly by affecting DCs and macrophages to produce IL-17 or IL-23 to interfere with the feed-forward mechanism.

In psoriasis lesions, IL-6 and IL-1β are released from stimulated keratinocytes. Together with TGF-β, IL-6 and IL-1β induce naive CD4^+^ T cells to differentiate into Th17 cells [51,52]. In addition, activated DCs and macrophages produce IL-6 to promote Th17-cell differentiation. Our results indicated that there was an increase in the amount and expression of IL-6 and IL-1β in the skins of IMQ model mice, but rTpp53 treatment significantly inhibited the increase in IL-6 in psoriatic skin lesions, suggesting that rTpp53 likely suppressed the Th17 response by inhibiting IL-6 and IL-1β production from various sources of cells.

The activated keratinocytes in psoriasis produce various cytokines, chemokines, and AMPs. AMPs have essential roles in skin immunity. More than 20 kinds of AMPs were found in human skin, some of which were highly expressed in psoriatic lesions, such as cathelicidin, beta-defensins, S100 proteins, and RNase [53]. In our present animal model, we tested several well-known AMPs, including S100a7, S100a8, beta-defensin 4 (Defb4), Defb14, lipocalin 2 (Lcn2) and cathelicidin antimicrobial peptide (Camp). S100A8, which is highly expressed in the lesion skin and serum of psoriasis patients [54], is produced by keratinocytes in the lesion skin and is thought to be involved in the pathogenesis of psoriasis via its chemotactic activity for neutrophils and CD4^+^ T lymphocytes [55]. The increase in *S100a8* expression in IMQ-induced psoriatic mice was most obvious, increasing more than 3000-fold compared with that in control mice. Increased expression was also observed in several tested AMPs, including *S100a7*, *Defb4*, *Defb14*, *Lcn2* and *Camp*. Surprisingly, the increased expression of all these AMPs was markedly suppressed by rTpp53 treatment in rTpp53+IMQ mice, suggesting that rTpp53 may directly affect keratinocytes to produce AMPs. The activation of keratinocytes also induces the production of various chemokines, such as CCL2, 5, 17, 20, 22 and CXCL1, 2, 3, 5, 8, 9, 10, 11. Chemokines are involved in the recruitment and activation of T lymphocytes, macrophages and neutrophils at the site of psoriatic inflammation and play important roles in the pathogenesis of psoriasis [56,57]. In our present IMQ-induced psoriatic mice, the expression levels of *Ccl2, 3, 12, and 20* and *Cxcl11, 12,* and *13* were increased (Fig. 4 and Table 1). Treatment with rTpp53 decreased the expression of these chemokines.

CCL20 plays an important role in the Th17 signalling pathway through its only ligand CCR6, which is a marker of Th17 cells. Therefore, CCL20 is thought to be an important factor in mediating the pathogenesis of psoriasis in the IL-23/IL17 axis [58,59]. CCL20 is expressed in normal human skin at low levels by epidermal keratinocytes, but its expression is abundantly increased in human psoriatic lesions, which are associated with proinflammatory cytokines, such as IL-17A, IL-23 and TNF-α [58]. In turn, IL-17A promotes keratinocytes to produce CCL20, and Th17 cells can also produce CCL20, which forms positive feedback in the pathogenesis of psoriasis. We tested the expression of *Ccl20* in psoriatic skin lesions and found an increase in expression in IMQ-induced psoriatic mice. The increased expression was downregulated by rTpp53 treatment, indicating that rTpp53 may suppress keratinocytes in the lesion site to produce CCL20, which leads to suppression of the Th17 response in IMQ mice.

In the IMQ model, the stimulation of IMQ not only causes immune responses in local skin lesions but also affects systemic immune responses, such as splenomegaly and increased responsiveness of splenocytes and macrophages. Treatment with rTpp53 in rTpp53+IMQ mice inhibited the increase in responsiveness, suggesting that the administration of rTpp53 to skin affects not only local but also systemic immune responses in vivo. Moreover, *in vitro* coculture of rTpp53 inhibited IL-17A production by splenocytes and IL-6 production by macrophages, suggesting that rTpp53 directly affects Th17 cells, DCs and macrophages.

The present study focused on the effect of the rTpp53 on IL-23/IL-17 signal pathway, because our previous study showed infection of *Trichinella* could inhibit Th17 response [2]. Although we found, in this study, rTpp53 to exert the suppressive effect on Th 17 response, it is necessary to further investigate its effect on psoriasis-related signal pathways such as NF-κB, TNF-α, JAK/STAT, Bruton’s tyrosine kinase, oxidation, and IL-2-inducible T-cell kinase-related signal pathways to reveal the detailed mechanism of the amelioration of psoriasis by rTpp53. Another point is that there is a limitation to using rTpp53 as a therapeutic agent for psoriasis because rTpp53 is a big molecular-weight protein with an unknown functional domain. For example, we used microneedles in this study to promote the absorption of rTpp53, it is still difficult to quantify rTpp53 in experimental samples and analyze pharmacokinetics (PK)/pharmacodynamics (PD). Therefore, further study is needed to determine the functional domain and/or epitope and establish a quantification method for rTpp53.

In conclusion, the 53 kDa ES protein from *T. pseudospiralis* ameliorates IMQ-induced psoriasis in an animal model. The amelioration is likely related to the inhibition of the Th17 response, as revealed by decreased expression of cytokines, chemokines, and AMPs in psoriatic lesions in the animal model. Our results provide new insights into parasitic immunomodulation through the Th17 signaling pathway. Our study also provides a new animal model of immune disorders to study parasite infection-induced immunomodulation and therapy potential for immune disorder diseases.

## Declaration of competing interest

The authors have no conflict of interest to declare that are relevant to the content of this article.
