# Supplementary material for: Trichinella pseudospiralis-secreted 53 kDa protein ameliorates imiquimod-induced psoriasis by inhibiting the IL-23/IL-17 axis in mice
Source: Biochem Biophys Rep. 2022 Dec 27;33:101415. doi: 10.1016/j.bbrep.2022.101415 (PMC9813687; doi:10.1016/j.bbrep.2022.101415)
Supplement: Multimedia component 1 [file mmc1.docx]

**Supplementary Table 1.** Primer for qPCR.

| **Gene** | **Sequence (forward/reverse)** | **Accession No.** |
| --- | --- | --- |
| *Gapdh* | ggcattgtggaagggctcat/gacacattgggggtaggaacac | NM_001289726.1 |
| *Ccl2* | cctgctgctactcattcacca/ccattccttcttggggtcag | NM_011333.3 |
| *Ccl3* | ctcccagccaggtgtcattt/ggcattcagttccaggtcag | NM_011337.2 |
| *Ccl12* | cacacttctatgcctcctgctc/ccggacgtgaatcttctgct | NM_011331.3 |
| *Ccl20* | cactcctggtggctctcg/gggtgacggatgtagtcctg | NM_001159738.1 |
| *Cxcl1* | tgcctgaacaccctaccaag/gagtggctatgacttctgtctgg | NM_008176.3 |
| *Cxcl2* | gcccagacagaagtcatagcc/ccaggtcagttagccttgcc | NM_009140.2 |
| *Cxcl5* | cggttccatctcgccatt/tccgttgcggctatgactg | NM_009141.3 |
| *Cxcl9* | ctggagcagtgtggagttcg/ggtctttgagggatttgtagtgg | NM_008599.4 |
| *Cxcl10* | ttttctgcctcatcctgctg/atggccctcattctcactgg | NM_021274.2 |
| *Cxcl13* | cctgaagccactatcatctcaa/gaggggagttattttcttggaa | NM_018866.2 |
| *Ccr5* | gaatgagaagaagaggcacagg/aggtggtcaggaggaggaca | NM_009917.5 |
| *Il1b* | gacggaccccaaaagatgaag/ctccacagccacaatgagtga | NM_008361.4 |
| *Il6* | ttccatccagttgccttcttg/catttccacgatttcccagag | NM_001314054.1 |
| *Il12b* | cagggacatcatcaaaccagac/gagaagtaggaatggggagtgc | NM_001303244.1 |
| *Il17a* | gtgtcaatgcggagggaaag/cctgaaagtgaaggggcag | NM_010552.3 |
| *Il17c* | gaaaccccgaagccatagga/cgagttagcaggtgtggagga | NM_145834.4 |
| *Il17f* | gtcgccattcagcaagaaatc/gcagccaacttttaggagca | NM_145856.2 |
| *Il23a* | tccagtgtgaagatggttgtga/gggctatcagggagtagagcag | NM_031252.2 |
| *Il36* | tggggaggtttttgacttgg/ggggttactctgtggcttcg | NM_153511.3 |
| *Tnfa* | ggcactcccccaaaagatg/cagtagacagaagagcgtggtg | NM_001278601.1 |
| *Mmp8* | ccttcctacccaacggtcttc/gcaggtcatagccacttagagc | NM_008611.4 |
| *Mmp13* | gctgcggttcactttgaga/tcttctatgaggcggggata | NM_008607.2 |
| *S100a7* | ggatagtgtgcctcgcttca/ctggttgtccttgtttttgtcag | NM_199422.1 |
| *S100a8* | tgccctctacaagaatgacttc/tttatcaccatcgcaaggaac | NM_013650.2 |
| *Defb4* | gtgctgctgtctccacttgc/tctgtcgaaaagcggtaggg | NM_019728.4 |
| *Defb14* | cttgttcttggtgcctgctc/cgacctatttgttcttcctttcc | NM_183026.2 |
| *Lcn2* | gtcgccattcagcaagaaatc/gcagccaacttttaggagca | NM_008491.1 |
| *Camp* | aagcagtgtatgggggcagt/cttgaaccgaaagggctgtg | NM_009921.2 |
